# Effect of antiresorbtive administration on the level of bone metabolism in osteoporosis

**DOI:** 10.1186/1753-6561-7-S1-P3

**Published:** 2013-01-30

**Authors:** Andreea Zoderu, Norin Forna, V Roxana

**Affiliations:** 1 Gr T Popa University of Medicine and Pharmacy, Iassy, Romania

## Background

Not long ago, osteoporosis was considered a normal condition specific to old age; presently, osteoporosis is an affection characterized through the reduction of the mineral bone density associated with the impairment of the trabecular bone structure and implicitly with the increase of the bone fragility. Compliance and adherence to osteoporosis management are of high priority, having a significant effect on the cost effectiveness of therapy 1.

## Methods

The evaluation of the phosphocalcium metabolism for the female patients with osteoporosis, before and after 1 year since the administration of antiresorptive drugs, was realized through osteodensitometry (BMD), that was the most conclusive paraclinical exam used in the diagnosis of osteoporosis, the evaluation of the clinical factors and the analysis of the bone turnover through the determination of the biochemical turn-over markers (BTM- bone formation and resorption, pre and post-therapy). Besides the specific analysis of these pathology was made blood analysis.

## Results

After 1 year since the administration of antiresorptive drugs wasn’t noticed important modifications at the BMD and biochemical markers, could contribute to the creation of a pathogenic profile of the patient that led to a therapeutic decision as correct as possible. BTM changes can also be used for understanding the mechanism of action of drugs in development and identifying the correct dose 2. Instead, at 2% of female patients could be noticed small decreases of the serum concentrations of calcium, as well as increases of the blood concentrations of the total activity of the alkaline phosphatase, without noticeable clinical consequences. Long-term alendronate administration may inhibit normal repair of microdamage arising from severe suppression of bone turnover (SSBT), which, in turn, results in accumulation of microdamage 3.

## Conclusions

The pharmacological therapy must be extended under periodical medical control in order to maximize the benefits and to avoid side effects since, depending on the results of the exams, it could be necessary to vary the doses or the type of used drug. The study of the hard support tissues is rendered difficult by the fact that the bone is one of the most unstable tissues from the organism.
